# Sexually Dimorphic Growth Stimulation in a Strain of Growth Hormone Transgenic Coho Salmon (*Oncorhynchus kisutch*)

**DOI:** 10.1007/s10126-020-10012-5

**Published:** 2021-01-22

**Authors:** Michelle T. T. Chan, Annette Muttray, Dionne Sakhrani, Krista Woodward, Jin-Hyoung Kim, Kris A. Christensen, Ben F. Koop, Robert H. Devlin

**Affiliations:** 1 Fisheries and Oceans Canada, 4160 Marine Drive, West Vancouver, BC V7V 1N6 Canada; 2 Present Address: Department of Molecular Biology and Biochemistry, Simon Fraser University, Burnaby, BC V5A 1S6 Canada; 3 Present Address: New York Institute of Technology, #1700-701 West Georgia Street, Vancouver, BC V7Y 1K8 Canada; 4 Present Address: Division of Life Sciences, Korea Polar Research Institute, 26 Sondomirae-ro, Yeonsu-gu, Incheon, 21990 South Korea; 5 Department of Biology, University of Victoria, Victoria, BC V8W 2Y2 Canada

**Keywords:** Sexually dimorphic growth, Transgenic coho, Growth hormone, Metallothionein-B, Estradiol

## Abstract

Growth hormone (GH) transgenic fish often exhibit remarkable transformations in growth rate and other phenotypes relative to wild-type. The 5750A transgenic coho salmon strain exhibits strong sexually dimorphic growth, with females possessing growth stimulation at a level typical of that seen for both sexes in other strains harbouring the same gene construct (e.g. M77), while males display a modest level of growth stimulation. GH mRNA levels were significantly higher in females than in males of the 5750A strain but equivalent in the M77 strain, indicating sex and transgene insertion locus altered transgene expression. We found that acute estradiol treatments did not influence GH expression in either strain (5750A and M77) or the transgene promoter (metallothionein-B), suggesting that estradiol level was not a significant factor influencing transgene activity. The feminization of XX and XY fish of the 5750A and M77 strains generated all-female groups and resulted in equalized growth of the two genetic sexes, suggesting that the presence of the Y chromosome was not directly capable of influencing the GH transgene–mediated growth in a physiological female conditions. These data suggest that the difference in growth rate seen between the sexes in the 5750A strain arises from non-estradiol-mediated sex influences on gene regulation at the transgene locus. This study shows how genetic factors and transgene insertion sites can influence transgene expression with significant consequent effects on phenotype.

**Supplementary Information:**

The online version contains supplementary material available at 10.1007/s10126-020-10012-5.

## Introduction

The successful development of transgenic strains for commercial use requires multiple generations to achieve and requires careful assessment of phenotype to ensure compatibility with production objectives. Significant changes in phenotype (e.g. physiology, morphology, and behaviour) of growth hormone (GH) transgenic fishes beyond growth acceleration have been observed, consistent with the pleiotropic nature of GH action on vertebrate physiology, morphology, and behaviour (Björnsson 1997; Devlin et al. 2015; Harvey et al. 1995). Under normal physiological conditions, secretion of GH from the pituitary stimulates production of IGF-1 in liver and other tissues, which in turn promotes growth and metabolism primarily by stimulation of the IGF-1 receptor (Wood et al. 2005). In transgenic fish, GH is expressed from multiple tissues and may act via endocrine and paracrine stimulation of GH receptors (Duan et al. 1993; Eppler et al. 2010; Hobbs and Fletcher 2008; Mori and Devlin 1999; Raven et al. 2008).

The majority of fast-growing transgenic salmon have been generated by non-homologous insertion of a heterospecific GH gene driven by a constitutively active promoter (Devlin et al. 1994; Du et al. 1992; Devlin et al. 2004). In GH transgenic fish, the magnitude of growth response elicited by GH overexpression can be influenced by genetic factors such as promoter strength, location of insertion, transgene copy number, and genetic background (Devlin et al. 2001; Leggatt et al. 2012; Lu et al. 1992; Martinez et al. 1999; Martinez et al. 2000; Rahman et al. 2000; Rahman and Maclean 1999; Sugiyama et al. 2012; Xu et al. 2013; Zhong et al. 2009; Devlin et al. 2004). For example, expression of GH transgene in the fast-growing domesticated strain of rainbow trout (*Oncorhynchus mykiss*) resulted in attenuated growth stimulation, but this was not observed in naturally slow-growing wild strain (Devlin et al. 2001). Genomic and endocrinological analyses revealed that, relative to wild-type, GH transgenesis and domestication affected GH and IGF-1 hormone levels as well as gene expression in similar ways (Devlin et al. 2009; Devlin et al. 2013; Fleming et al. 2002; Tymchuk et al. 2009). Despite parallel responses, genome-wide association studies (GWAS) have found that loci influencing growth and gene expression levels are highly distinct between GH transgenic and wild-type salmon (Kodama et al. 2018b; Mcclelland et al. 2020). The degree of growth enhancement is also influenced by environmental conditions and genotype-by-environment interactions. For example the effect of a transgene on growth was very different between fish grown in tank vs. stream environments (Sundström et al. 2007). Together, these data show that the effect of a transgene is context specific, being influenced by both environmental background and genetic background.

The presence or absence of the Y chromosome represents a major difference in genetic background among individuals that results in alternate reproductive endocrinology, growth, gonadal development, and sexually dimorphic morphologies. However, in salmonids, these differences are primarily associated with later stages of development as a result of sexual selection (Garcia De Leaniz et al. 2007; Gutierrez et al. 2015; Kodama et al. 2018a; Taranger et al. 2010). Sex-dependent growth difference is often difficult to detect in juvenile salmon due to family, temperature, and light effects; if detected, males are often found to grow faster than females (Kodama et al. 2018a; Martin-Smith and Armstrong 2002; Yamamoto 2004; Mizzau et al. 2013). For multiple GH transgenic coho salmon (*Oncorhynchus kisutch*) strains showing strong growth enhancement, sexually dimorphic growth has not been detected (Leggatt et al. 2012; Devlin et al. 2004). During the development of GH transgenic coho salmon strains, one unusual line, 5750A, was found to stimulate growth differently between the sexes. Growth stimulation in females was at a level typical of that seen in other GH transgenic strains made with the same gene construct and in the same genetic background, whereas males showed a reduced stimulation of growth relative to their transgenic female siblings.

To understand the interaction between the transgenic locus with the genome, we compare the differential growth response between sexes in two strains, one lacking sexually dimorphic growth stimulation (strain M77) and the other showing reduced growth stimulation in males relative to females (5750A). In the 5750A line, the transgene was previously found at a single telomeric locus of the acrocentric chromosome, whereas the transgene of the M77 strain was found at a single centromeric locus of a metacentric chromosome (Phillips and Devlin 2009). Here, we examine whether the difference in growth stimulation seen between sexes and strains is due to the presence/absence of the Y chromosome or the reproductive physiology status of the animal (using estradiol feminization of XY males), and assess whether GH and IGF-1 gene expression are differentially regulated between the sexes in the two strains and are correlated with the degree of growth stimulation.

## Materials and Methods

### Fish Culture and Sampling

Two GH transgenic coho salmon (*O. kisutch*) strains (M77 and 5750A) containing the OnMTGH1 growth hormone gene construct were utilized. This construct possesses a salmon metallothionein-B (MT-B) promoter driving expression of the full-length salmon GH1 gene. In the M77 strain, GH has been shown to be overexpressed in all tissues examined (Mori and Devlin 1999), and results in the stimulation of IGF-1 expression (Raven et al. 2008) and elevated growth. In the course of transgenic strain development (Devlin et al. 2004), males from 5750A strain predominately matured at the normal 3–4 years of age with a typical size of wild-type fish in culture conditions, rather than as 2 years old typical of GH transgenic females of the same strain and both sexes of other transgenic strains.

To assess the growth of M77 and 5750A males and females in controlled crosses, four crosses between transgenic and wild-type salmon were made for each strain, specifically, two hemizygous transgenic female × wild-type male crosses and two wild-type female × hemizygous transgenic male crosses. One 5750A cross had extremely poor viability due to poor egg quality from the wild-type mother and consequently this family was excluded from further analysis. To equalize examination of strains within the study, one randomly selected family was also eliminated. Newly feeding fry from each family were raised in separate 200-L tanks in 10 °C well water under a simulated natural photoperiod. Fish were fed with commercial salmon feed (Skretting Canada) to satiation between 3 and 8 times daily depending on developmental stage. All procedures and handling of the animals were conducted under permit AUP-12-003 from the DFO Pacific Region Animal Care Committee following Canadian Council on Animal Care guidelines.

In May, 48 first-feeding fry from each family were randomly selected to measure size and to genotype for sex and transgene presence as described earlier. A further random sample of 200 fry was sampled monthly for size and genotype starting in July and continuing until October. During the third sampling (September), transgenic individuals from each family were anaesthetized with 70 mg tricaine methanesulphonate (TMS) per litre buffered with 140 mg sodium bicarbonate per litre and tagged intraperitoneally with a passive integrated transponder (PIT) and pooled into a 3000-L common-garden tank. Because there were no significant differences in size between males and females of non-transgenic coho salmon in each family, a control group was established comprised of 20 non-transgenic coho salmon from each family that were randomly chosen and pooled together in one 200-L tank.

### Acute Treatment of 5750A and M77 Transgenic Coho Salmon with Estradiol

To examine whether M77 and 5750A strains showed different transgene expression levels between sexes, RT-PCR was performed to measure the expression of liver GH mRNA as well as its downstream effector IGF-1 (see ‘Molecular Biology’). We acutely treated both strains by intraperitoneal injection of estradiol at a dose of 5 mg per kilogramme body weight as described in Korte et al. (2000). The injection mixture comprised of 5 mg of estradiol (Sigma) dissolved in 10 μL of ethanol before being mixed at a 1:9 ratio with corn oil. Control treatments lacked estradiol. The mixture was then emulsified through vortexing before intraperitoneal injection. Prior to injection, fish were anaesthetized with TMS as described earlier. As a control for treatment efficacy, we confirmed that this estradiol dosage effectively induces the estradiol-responsive gene vitellogenin (Vtg; Supplementary Figure 1). Fish were grown for an additional 6 weeks post-injection before being euthanized for liver tissue collections for RNA extraction.

### Molecular Biology

RNA extraction from hepatic tissues, cDNA synthesis, and quantification of GH, IGF-1, Vtg, and ubiquitin by qPCR were performed as described previously (Raven et al. 2008). Briefly, each reaction utilized 12.5 ng of cDNA and either Fast SYBR green master mix (Applied Biosystems) for detection of ubiquitin, or using TaqMan probes and the TaqMan fast universal PCR master (Supplementary Table 1). Amplification was performed on a 7500 Fast Real-time PCR System (Applied Biosystems). Relative transcript levels were calculated through the ΔΔCt method, using ubiquitin as the reference transcript (Livak and Schmittgen 2001). Ubiquitin levels were statistically equivalent between the treatments, with the exception of the estradiol-treated males of the 5750A strain (data not shown). The difference between the mean of the untreated M77 females, which was the group with the highest ubiquitin expression level, and the mean of the 5750A estradiol-treated males was 8.6% (ANOVA, *p* < 0.01). As this difference was small, ubiquitin was used as a reference gene. The lowest untreated M77 sample was chosen as the reference sample, where its expression was set to a value of 1, with other samples normalized to it to generate relative mRNA measures. Expression of the positive control gene Vtg was assessed to confirm that estradiol treatments were effective. Vtg is a biomarker for estrogenic compound exposure in male fish.

### Sex Reversal Growth Trial of Males and Females of Transgenic and Non-transgenic 5750A Coho Salmon Using Estradiol

To assess whether the observed differential growth between female and male 5750A transgenic salmon was a result of physiological sex or genotypic sex, male fish were feminized using treatments with estradiol (Devlin and Nagahama 2002; Hunter et al. 1983; Piferrer and Donaldson 1992) to generate genetic (XY) females. Treatments consisted of immersing newly hatched alevin in water containing estradiol at a concentration of 400 mg/L for 2 h and repeating once a week for 4 weeks until they reached the fry stage. Subsequently, fry were given feed with added estradiol (10 mg of estradiol per kg of feed) for 60 days. After a growth period of 118 days, fish were genotyped, examined visually for the development of ovaries, and were genetically sexed by PCR to distinguish genetic males (XY) and genetic females (XX). All transgenic fish (*N* = 20) were found to be feminized (presence of ovary visually confirmed), whereas 26/29 non-transgenic fish were feminized (the 3 non-feminized fish were removed from analyses). Attempts made to masculinize the 5750A females with methyl-testosterone following conditions described in Piferrer and Donaldson (1992) were unsuccessful, as treated genetic (XX) females still developed ovaries.

### Statistical Analysis

For the initial growth trial, relationships among body weights at the October sampling point were assessed among strains and between sexes within a strain using a two-way ANOVA. Some groups did not possess equal variance or possessed distributions that could not be normalized by transformation, and thus non-parametric Mann-Whitney rank sum tests were also performed to assess body size between sexes within families (SigmaPlot, SAS). For the sex reversal trial, a Shapiro-Wilk test of normality was performed in GraphPad Prism (version 5) to ensure that data was normally distributed prior to ANOVA to determine whether significant differences existed between treatment groups at a significance level of *α* = 0.05. Gene expression data were assessed by two-way ANOVA in XLSTAT (Addinsoft) between strains and among strains based on sex and treatment in XLSTAT using log-transformed data.

## Results and Discussion

### Growth Between Sexes and Strains

Typically, no differences in growth rate between males and females of the M77 and H3 strains of GH transgenic coho salmon have previously been noted (Leggatt et al. 2012; Devlin et al. 2012). However, preliminary observations of the 5750A strain suggested that males did not grow at the same fast rate seen for females of the same strain and for both sexes of other strains. To accurately assess these observations, a growth trial was conducted in which the 5750A strain was grown alongside the M77 strain of GH transgenic coho salmon which had shown no noticeable difference in the growth of males and females over previous generations.

**Fig. 1:**
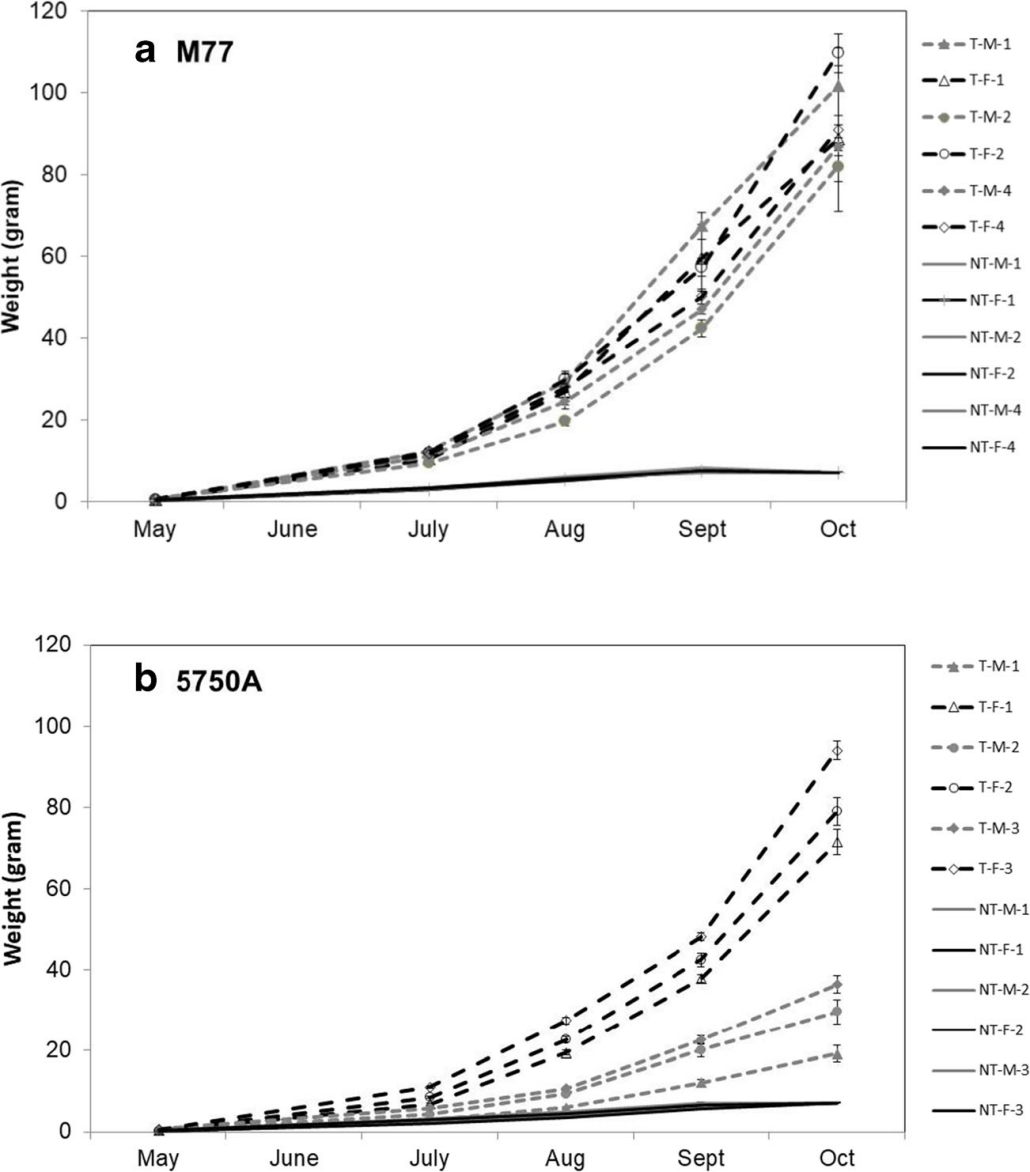
Weights of three male (M) and female (F) transgenic (T) and non-transgenic (NT) back-crossed families measured over a 5-month period. Each point represents the mean weight and the bars represent standard error. (A) M77 strain and (B) 5750A strain of coho salmon

Male and female transgenic fish from the M77 and 5750A strains both outgrew their non-transgenic siblings in weight (Fig. 1A and B, respectively) and length (not shown). While both strains showed an elevated growth rate compared to non-transgenic salmon, no overall difference in growth was detected between the sexes for M77 strain as there was a statistically significant interaction between family and sex (*p* < 0.001) (Fig. 1A). Only one of three transgenic M77 families showed a significant difference between weight of females to males at final sampling (family 1, *p* = 0.915; family 2, *p* < 0.001; family 3, *p* = 0.211). In contrast, the 5750A families had a consistent sex-specific difference in which the growth of the females was significantly greater than that of the males (Fig. 1B) (*p* < 0.001) with no statistically significant interaction between sex and family (*p* = 0.374). Females from strain 5750A possessed rapid growth equivalent to both sexes of strain M77 (Fig. 1A and B), whereas growth of males from strain 5750A was slower. Differences in growth between 5750A males and females could be detected as early as 4 months after hatching (*p* < 0.01), and persisted into adulthood (Fig. 1B). Slow-growing transgenic males and fast-growing females were produced from crosses of a non-transgenic parent to either a transgenic dam or a transgenic sire, indicating the sexual dimorphism in 5750A does not follow a sex-linked inheritance pattern and does not depend on maternal and paternal inheritance. Reciprocal crosses between 5750A transgenic and wild-type coho salmon followed by PCR genotyping (Fitzpatrick et al. 2011) and genetic and phenotypic sex identification (Muttray et al. 2017) revealed that the transgene insert was not sex linked (data not shown). At the final sampling point in October, the ratio of the mean male to female weight (0.71) in strain M77 was less than that for each of the 5750A families (male to female weight ratios: family 1 = 0.27; family 2 = 0.37; family 3 = 0.39; average = 0.34).

Although there was obvious sexually dimorphic growth of the transgenic 5750A strain, we also observed a small number of male individuals that grew at rates as females. The frequency distribution of fish weights at 5 months (Supplemental Figure 2 A, B, C) revealed M77 transgenic males and transgenic females were located in the same unimodal distribution. In contrast, a bimodal distribution of transgenic fish was segregated for the most-part by sex in strain 5750A, but there were a small number of transgenic males that had growth similar to their transgenic female siblings. We do not currently understand the mechanistic basis for how some males do not show growth suppression as seen for the majority of males. However, as both male and female 5750A transgenic salmon can produce highly growth stimulated females and slower-growing males suggests that the transgenic locus is capable of high levels of growth stimulation under some conditions even in the same sex, and as such is not due to a fixed condition associated with the structure of the transgene insert between males vs. females. It is known that transgenic loci in fish can show variable mosaic expression among individuals within lines (Martin and Mcgowan 1995; Mcgowan and Martin 1997; Rahman et al. 2000) that can be affected by genetic modifiers and by external environmental conditions, e.g. temperature or nutrient reserves of the yolk (Martin and Mcgowan 1995; Mizzau et al. 2013). It is possible that such epigenetic factors may be influencing the expression of the 5750A transgene such that expression of the transgene may be suppressed in the majority of males relative to females (with XY vs. XX genotypes, respectively), but that some males can escape such suppression depending on their background genotype and environmental conditions they experience.

### Expression of GH and IGF-1 Following Acute Estradiol Treatment

**Fig. 2:**
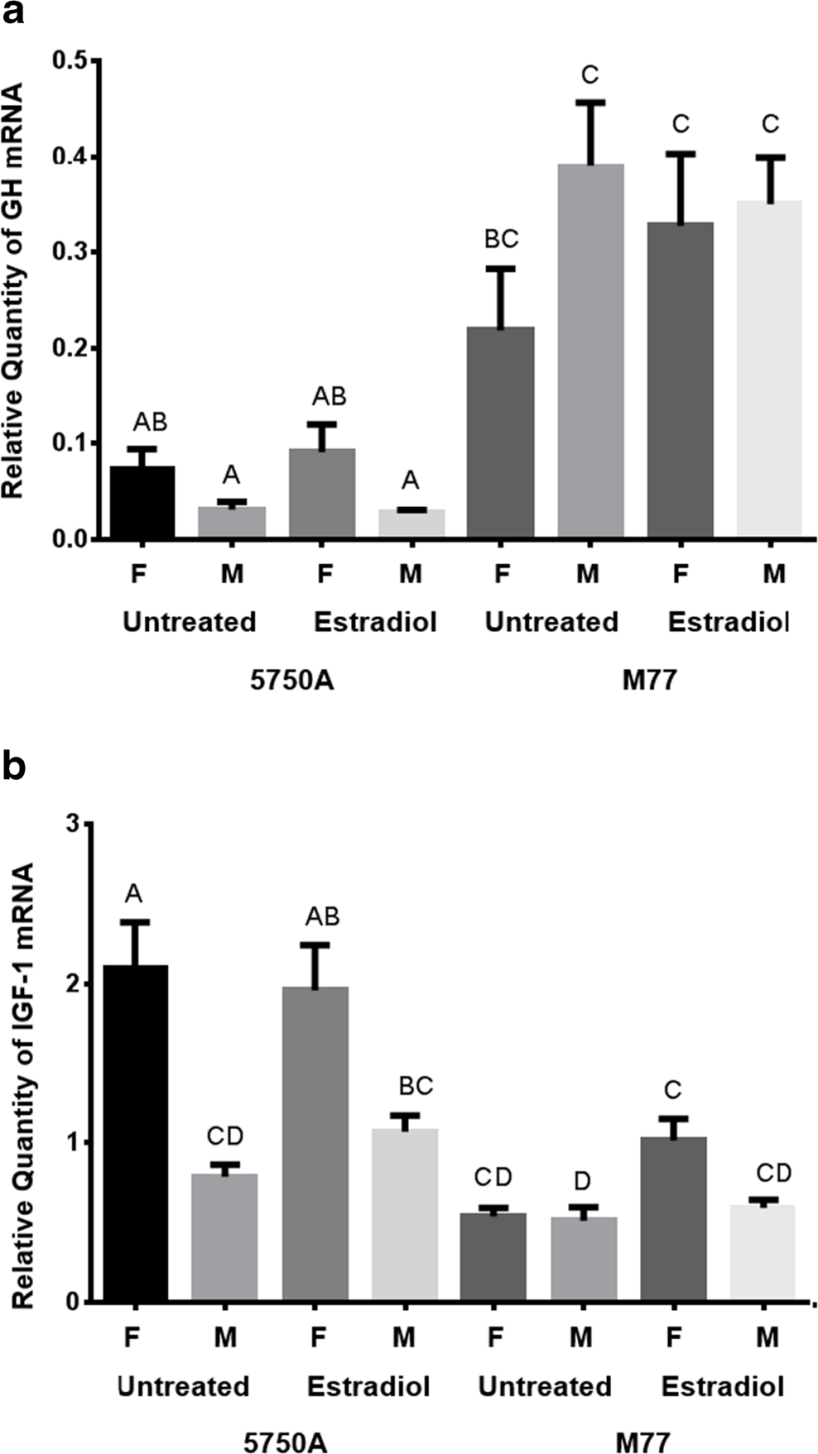
Relative GH (A) and IGF-1 (B) mRNA levels from liver samples of estradiol-treated and untreated males (M) and females (F) of the 5750A and M77 strains of GH transgenic coho salmon. The bars represent the mean ± standard error. *N* = 9–18. Capital letters above the bars (A, B, C, D) represent statistically significant differences between the means

Female physiology and male physiology differ in multiple ways that have the potential to affect growth (Duan et al. 1994; Fukada et al. 2003), but whether these influences operate in the same fashion in GH transgenic fish is not known. In the present context, we examined the expression of GH and its downstream effector IGF-1 in males and females in GH transgenic strains M77 and 5750A. We treated the strains with intraperitoneal injection of estradiol to assess whether this major sex hormone was able to influence the expression of the transgene. A positive control for hormone treatment was performed and found that expression of the estradiol-regulated gene Vtg was highly induced under the experimental conditions (Supplemental Figure 1), inducing Vtg 7.6 × 10^2^ fold in 5750A males and 8.7 × 10^1^ fold in 5750A females over non-treated fish. Induction was even higher in M77, 3.5 × 10^4^ fold in males and 7.1 × 10^3^ fold in females. The significant difference in Vtg expression levels between estradiol-treated and non-treated fish (*p* < 0.0001) confirmed that the feminization through estradiol treatments was successful in inducing the expression of female sex hormones. GH mRNA levels were greater in M77 than in 5750A (Fig. 2A, *p* < 0.001), suggesting stronger expression of the GH transgene in the former strain. No effects of genetic sex nor estradiol treatment were apparent in strain M77. However, in strain 5750A, GH mRNA levels were significantly higher (two-way ANOVA, *p* = 0.026) in females than in males (assessing treated and untreated fish together), indicating female sex was affecting liver transgene expression in this strain. However, estradiol treatment did not significantly change the GH expression pattern either between sexes or between strains, suggesting that at least this level or duration of sex steroid treatment is not a major factor responsible for the transgene expression differences seen between the sexes in strain 5750A.

The pattern of IGF-1 mRNA expression in strain 5750A was found to be similar to the pattern of GH expression, with genetic females possessing higher levels than genetic males (two-way ANOVA, *p* < 0.0001), and no influence of estradiol treatment. Levels of IGF-1 in strain M77 did not differ by sex or estradiol treatment, but overall levels of IGF-1 were lower than in strain 5750A (Wilk’s test, *p* < 0.0001). Thus, there appears to be an inverse relationship between GH and IGF-1 levels, with low GH and high IGF-1 being seen in strain 5750A and vice versa for strain M77, and a relatively poor relationship with growth at the strain level. It is possible that low levels of expression of the 5750A transgene may not be saturating GH receptors and can thus elicit differential mRNA levels for IGF-1 between the sexes, whereas for strain M77, high levels of GH expression from the transgene may saturate GH receptors and thus may prevent sex-related influences to be dampened and not able to influence growth. In strain M77, GH has been shown to be overexpressed in all tissues examined (Mori and Devlin 1999), and correlates with stimulation of IGF-1 expression in similar tissues (Raven et al. 2008). Sex differences in expression could arise by differential activity of the MT-B promoter that drives expression of GH from the transgene. Thus, we examined whether the MT-B gene in the coho salmon genome showed a difference in expression between sexes and in response to estradiol. Higher expression of metallothionein has been reported in female mice compared to male mice (Zhang et al. 2012). Conversely, Gerpe et al. (2000) have shown an indirect reduction of MT expression after estradiol injection in Arctic char. Although strain 5750A had higher levels of MT-B expression than M77 overall, no differences between sexes were detected (Supplemental Figure 3), suggesting the difference in transgene expression between male and female in 5750A is specific to the transgene insert locus rather than as an indirect property of the MT-B promoter driving the transgene. No effect of estradiol treatment on MT-B expression was observed, indicating that it is not female steroid physiology that causes differential regulation of the GH transgene in strain 5750A between the sexes, but rather it is caused by a separate difference in sex physiology.

### Growth of 5750A Transgenic Feminized Males

**Fig. 3:**
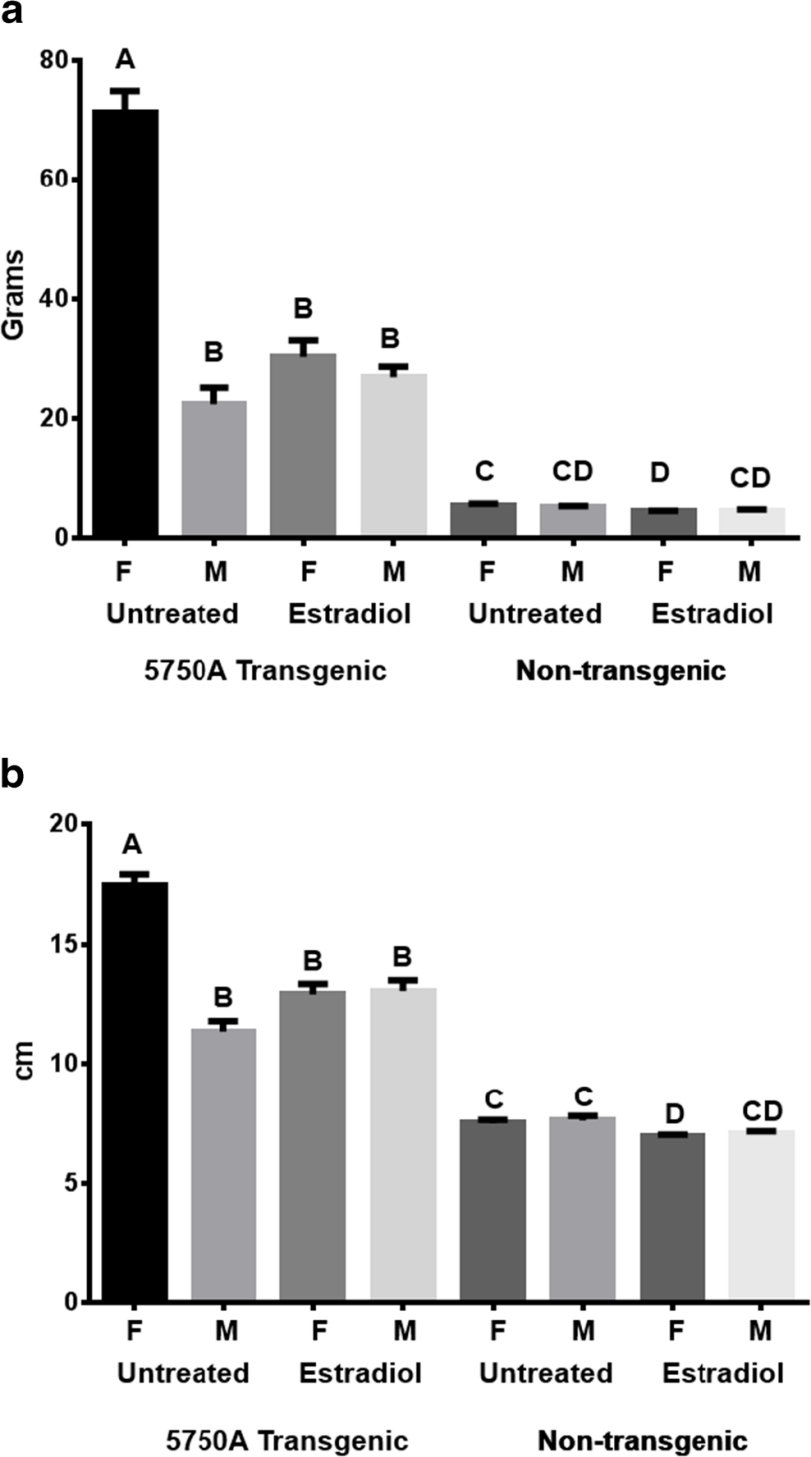
Weight (A) and length (B) of 5750A GH transgenic and non-transgenic coho salmon with or without estradiol treatment measured after an 8-week growth period. Estradiol treatment caused feminization of male fishes in both families. Bars are mean ± standard error, and *n* = 6–40. F, female; M, males; capital letters above the bars (A, B, C, D) indicate significant differences between means

Our data show that the 5750A transgene elevates growth in a strongly sex-dependent fashion. It is possible this difference arises from genetic differences between males and females (XY vs. XX) or is due to secondary physiological differences (e.g. sex steroids). Expression of the primary sex-determination gene, sdY, in salmon (Yano et al. 2013) initiates a cascade of physiological differences between the sexes that likely arise from the differential expression of many genes in the sex-determination pathway. It is conceivable that the 5750A transgene insertion site is within or adjacent to a locus that is regulated by sex, and that the transgene’s expression is affected by the local chromosomal regulatory domain. It is also possible that the transgene is directly affected by the regulatory influences of the Y chromosome that are independent of the physiological sexual state of the individual. To examine these possibilities, we examined the effect of feminization on growth of XY vs. XX genetic sexes in strain 5750A. No significant difference in weight or length was observed between the sexes in the wild-type (non-transgenic) coho salmon, indicating that under our experimental conditions, dimorphic growth between the sexes at this stage of development was not apparent (Fig. 3A). However, feminization treatment with estradiol was found to reduce both weight and length of XX (and length of XY) non-transgenic salmon (Fig. 3 A and B). Similarly, for transgenic 5750A XX coho salmon, treatment with estradiol reduced growth for both weight and length. These data are consistent with previous studies showing the growth-suppressive properties of estrogenic compounds in non-transgenic fish (Hanson et al. 2014; Ostrowski and Garling 1986). Untreated 5750A XY males were smaller than their female siblings (as described earlier for this strain), whereas both XY and XX females were the same size (i.e. XY females did not show a further reduction in growth relative to XY males). Thus, these differential responses to estradiol treatment, feminization, and sex-chromosome genotype have an overall effect of equalizing the growth between the two genetic sexes (XY and XX) in transgenic 5750A salmon, and suggest that the presence/absence of the Y chromosome does not directly affect growth in this strain. Alternatively, the physiological female condition (possessed in estradiol-treated XX females and in estradiol-treated XY males) eliminates the sexually dimorphic growth-enhancing effects of the 5750A transgene. Regardless of whether the 5750A transgenic coho salmon is genetically male or female, the presence of an ovary and presumably expression of female-related sex-biased genes appears to equalize the growth between sexes. However, that non-transgenic XX females and XY males do not show a growth difference in contrast to the 5750A strain suggests that some differences in sex-regulated genes likely exist in juvenile salmon.

## Conclusions

The dimorphic growth between female and male observed in the transgenic coho salmon strain 5750A, but not in strain M77, demonstrates that transgene expression is influenced by the location of transgene insertion and is under the effect of genetic background, in this case presence/absence of the Y chromosome (male vs. female sex). Here, we showed that acute treatments with estradiol did not cause different regulatory effects on growth, whereas chronic treatments that resulted in all-female groups eliminated the observed dimorphic growth patterns in GH transgenic 5750A suggestive of locus-dependent transgene regulation. We speculate that the different insertion sites between the two strains may have differing transcriptional regulatory environments that could influence transgene expression between the sexes. Further studies into epigenetic controls of the transgene are required to elucidate mechanisms behind dimorphic growth seen in this unusual GH transgenic coho salmon strain.

## Supplementary Information

Supplementary Table 1 (DOCX 27 kb)

Supplementary Figure 1 (DOCX 36 kb)

Supplementary Figure 2 (DOCX 79 kb)

Supplementary Figure 3 (DOCX 104 kb)

## Data Availability

Not applicable
