# Supplementary material for: Sexually Dimorphic Growth Stimulation in a Strain of Growth Hormone Transgenic Coho Salmon (Oncorhynchus kisutch)
Source: Mar Biotechnol (NY). 2021 Jan 22;23(1):140–8. doi: 10.1007/s10126-020-10012-5 (PMC7929968; doi:10.1007/s10126-020-10012-5)
Supplement: Supplementary file 1 — (DOCX 27 kb) [file 10126_2020_10012_MOESM1_ESM.docx]

Supplemental Table 1. Primer and Probe sequences used in RT-qPCR

| **Primer/Probe** | **Sequence (5'-3')** |
| --- | --- |
| Ubiquitin Forward Primer | CAACAGCGTCTGATCTTCGC |
| Ubiquitin Reverse Primer | TTTGTCACAGTTGTACTTCTGGGC |
| GH Forward Primer | CAAGATATTCCTGCTGGACTTCTGT |
| GH Reverse Primer | GGGTACTCCCAGGATTCAATCA |
| GH-FAM Probe | CAGTCCTGAAGCTGC |
| IGF-1 Forward Primer | GGCATTTATGTGATGTCTTCAAGAGT |
| IGF-1 Reverse Primer | CCTGTTGCCGCCGAAGT |
| IGF-1-FAM Probe  VTG Forward Primer*  VTG Reverse Primer*  VTG-FAM Probe* | TCTCACTGCTGCTGTGC  CTGCCAGCAGAGAATCATGAAG  CTGCCGGCACTCTACACACTT  CTTTGGTCTGGCTTACAC |

^*^ Vtg primers and probe were designed from alignments of multiple vitellogenin sequences in Clustal.
