# Supplementary material for: Sexually Dimorphic Growth Stimulation in a Strain of Growth Hormone Transgenic Coho Salmon (Oncorhynchus kisutch)
Source: Mar Biotechnol (NY). 2021 Jan 22;23(1):140–8. doi: 10.1007/s10126-020-10012-5 (PMC7929968; doi:10.1007/s10126-020-10012-5)
Supplement: Supplementary file 2 — (DOCX 36 kb) [file 10126_2020_10012_MOESM2_ESM.docx]

C

C

C

C

B

A

A

AB

**Supplemental Figure 1** Treatment with estradiol elicits an estrogenic response in both M77 and 5750A GH-transgenic *O.kisutch* strains, inducing vitellogenin 7.6 x 10^2^ fold in 5750A males and 8.7 x 10^1^ fold in 5750A females, and 7.4 x 10^4^ fold in M77males and 7.1 x 10^3^ fold in M77 females. This confirms that the feminization through estradiol treatments was successful in inducing the expression of female sex hormones.
