# Supplementary material for: Sexually Dimorphic Growth Stimulation in a Strain of Growth Hormone Transgenic Coho Salmon (Oncorhynchus kisutch)
Source: Mar Biotechnol (NY). 2021 Jan 22;23(1):140–8. doi: 10.1007/s10126-020-10012-5 (PMC7929968; doi:10.1007/s10126-020-10012-5)
Supplement: Supplementary file 3 — (DOCX 79 kb) [file 10126_2020_10012_MOESM3_ESM.docx]

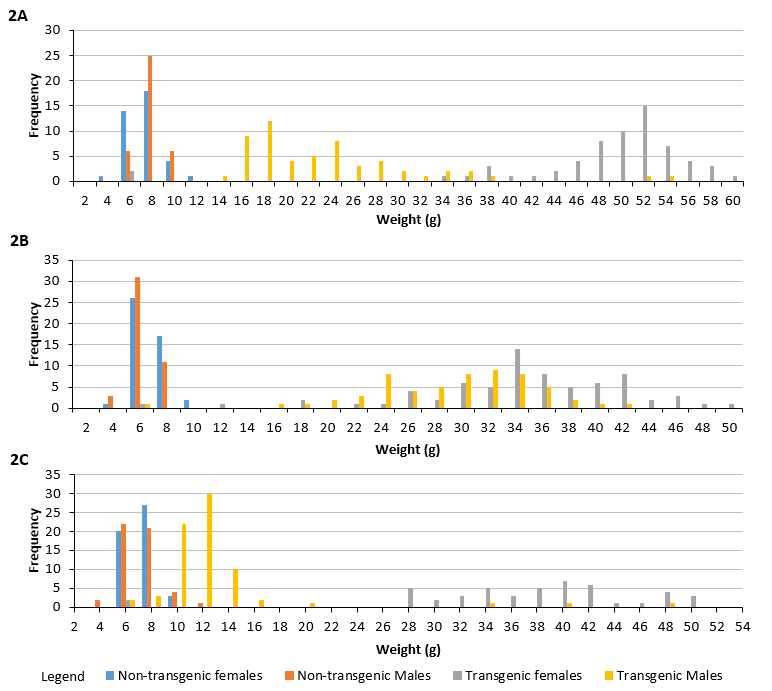


Supplemental Figure 2 Frequency distribution of weight at 5 months of age of A) a 5750A family created from a 5750A sire and non-transgenic dam, B) a M77 family created from a non-transgenic sire and M77 dam, C) a 5750A family created from a non-transgenic male and 5750A dam, this family exhibited overlap in size between transgenic males and non-transgenic male and females.
