# Supplementary material for: Sexually Dimorphic Growth Stimulation in a Strain of Growth Hormone Transgenic Coho Salmon (Oncorhynchus kisutch)
Source: Mar Biotechnol (NY). 2021 Jan 22;23(1):140–8. doi: 10.1007/s10126-020-10012-5 (PMC7929968; doi:10.1007/s10126-020-10012-5)
Supplement: Supplementary file 4 — (DOCX 104 kb) [file 10126_2020_10012_MOESM4_ESM.docx]

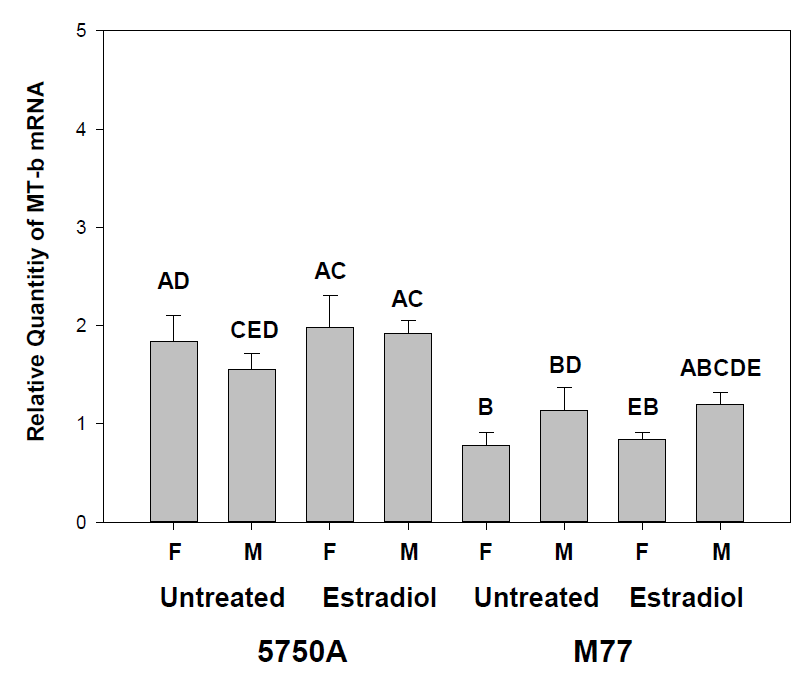


**Supplemental Figure 3** Relative metallothionein-b (MT-b) mRNA levels from liver samples of estradiol treated and untreated males (M) and females (F) of the 5750A and M77 strains of GH transgenic coho salmon. The bars represent the mean ± standard error. Capital letters above the bars (A, B, C, D) represent statistically significant differences between the means.
